# Comparison of three cutaneous antiseptic solutions for the prevention of catheter colonization in an ICU for adult patients: a multicenter prospective randomized controlled trial

**DOI:** 10.1186/cc14153

**Published:** 2015-03-16

**Authors:** H Yasuda, M Sanui, T Komuro, J Hatakeyama, S Matsukubo, S Kawano, H Yamamoto, K Andoh, R Seo, N Shime, E Noda, N Saito

**Affiliations:** 1 Japanese Red Cross Musashino Hospital, Tokyo, Japan; 2 Saitama Medical Center Jichi Medical University, Saitama, Japan; 3 YokohamaCity Minato Red Cross Hospital, Kanagawa, Japan; 4 Kurashiki Central Hospital, Okayama, Japan; 5 The Jikei University School of Medicine, Tokyo, Japan; 6 Toyonaka Municipal Hospital, Osaka, Japan; 7 Sendai City Hospital, Miyagi, Japan; 8 Kobe City Medical Center General Hospital, Hyogo, Japan; 9 National Hospital Organization Kyoto Medical Center, Kyoto, Japan; 10 Kyushu University Hospital, Fukuoka, Japan; 11 Nippon Medical School Chiba Hokusoh Hospital, Tiba, Japan

## Introduction

The current CDC guideline for the prevention of intravascular catheter-related infections recommends skin preparation with a greater than 0.5% chlorhexidine with alcohol solution before central venous catheter (CVC) or peripheral arterial catheter (AC) placement. However, few studies investigated the superiority of 1% alcoholic chlorhexidine gluconate (1% CHG) over either 0.5% alcoholic chlorhexidine gluconate (0.5% CHG) or 10% aqueous povidone iodine (10% PVI) for the prevention of catheter colonization. The aim of this study is to compare the effectiveness of three skin antiseptic solutions for the prevention of intravascular catheter colonization.

## Methods

This multicenter prospective randomized controlled trial was conducted in 15 Japanese ICUs from December 2012 to March 2014. Patients over 18 years of age undergoing CVC and AC placement in the ICU are randomized to have one of three skin antiseptic preparations before catheter insertion. After removal of the catheter, the distal tip is cultured using semiquantitative or quantitative techniques. The incidence of catheter colonization and catheter-related bloodstream infection (CRBSI) is compared between the three groups.

## Results

A total of 997 catheters were placed, including 339 catheters using 1% CHG, 329 using 0.5% CHG, and 329 using 10% PVI. The median duration of catheter indwelling in the entire population was3.7 days with an interquartile range of 2.0 to 6.7 days, with no significant difference between the groups (*P *= 0.36). Thirteen catheters (5.1%) in the 10% PVI group were positive for catheter-tip colonization, whereas six catheters (2.2%) in the 1% CHG group and five catheters (1.9%) in the 0.5% CHG group were positive (*P *= 0.07). The probability of catheter colonization was significantly higher in the 10% PVI group than each CHG groups (*P *= 0.028, log-rank test). The incidence of catheter colonization and CRBSI is compared between the three groups.

## Conclusion

In this randomized controlled trial comparing the effectiveness of three cutaneous antiseptic solutions for the prevention of catheter colonization, either 0.5% or 1.0% CHG was superior to 10% PVI.

